# Sig1R activates extracellular matrix-induced bladder cancer cell proliferation and angiogenesis by combing β-integrin

**DOI:** 10.18632/aging.204721

**Published:** 2023-05-16

**Authors:** Feng Zhao, Tianli Yang, Liuhua Zhou, Rongfei Li, Jingyu Liu, Jun Zhao, Ruipeng Jia

**Affiliations:** 1 Department of Urology, Nanjing First Hospital, Nanjing Medical University, Nanjing 210006, China

**Keywords:** bladder cancer, ECM, Sig1R, β-integrin, ion channel

## Abstract

The extracellular matrix (ECM) regulates many biological functions involved in tumorigenesis and tumor development; however, the underlying mechanism remains unknown. Sigma 1 receptor (Sig1R), a stress-activated chaperone, regulates the crosstalk between the ECM and tumor cells and is related to the malignant characteristics of several tumors. However, the link between Sig1R overexpression and ECM during malignancy has not been established in bladder cancer (BC).

Here, we analyzed the interaction of Sig1R and β-integrin in BC cells and its role in ECM-mediated cell proliferation and angiogenesis. We found that Sig1R forms a complex with β-integrin to promote ECM-mediated BC cell proliferation and angiogenesis, which enhances the aggressiveness of the tumor cells. This leads to poor survival. Our research revealed that Sig1R mediates the cross-talk between BC cells and their ECM microenvironment, thereby driving the progression of BC. Promisingly, targeting an ion channel function through Sig1R inhibition may serve as a potential approach for BC treatment.

## INTRODUCTION

Bladder cancer is a common urologic malignant tumor that ranks fifth among the common tumors [1, 2]. Approximately 430, 000 new bladder cancer cases and 165, 000 deaths related to bladder cancer have occurred globally [1, 3]. The early stage of bladder cancer typically has no obvious clinical symptoms. Once the patient has hematuria, dysuria, or other symptoms, this indicates that the development of the tumor has entered a late stage. Studies show that 25-30% of bladder cancer patients have muscle infiltration at the time of initial diagnosis, and 15% have local or distant metastasis with poor prognosis [4–6]. The recurrence rate of non-muscle-infiltrating BC was as high as 70% within 5 years, and 10-20% of cases eventually progress to muscle-infiltrating BC [7, 8]. Therefore, there is an urgent need to identify new sensitive and specific tumor biomarkers for early detection and treatment.

The extracellular matrix (ECM) is the acellular compartment of an organism and is mainly composed of proteoglycans and fibrous proteins [9]. ECM regulates cell shape, polarity, cell apoptosis, proliferation, and invasion, thus exerting biomechanical properties [9, 10]. Studies have found that an imbalance in the ECM and abnormal remodeling are key events in tumor appearance and progression. The specific arrangement and direction of ECM components form a tissue-specific microenvironment that regulates the behavior of tumor cells by inducing tumor-related genes [11]. Studies have shown that small changes in the ECM microenvironment can promote tumor progression by interfering with cell adhesion and polarity and by amplifying growth factor signaling [11, 12].

Sigma 1 receptor (Sig1R) is a 25 kDa stress-activated chaperone protein anchored at the endoplasmic reticulum-mitochondria interface. It is involved in neurodegenerative diseases and stroke and is also associated with cocaine addiction [13–15]. Although Sig1R is highly expressed in many tumor cells and is involved in their regulation, there is very limited research on the protein [16–18]. According to previous reports, Sig1R act as a chaperone to promote voltage-gated K^+^ channel maturation and interact with multiple ion channels, including biosensors that participate in the communication between cells and the ECM [19–21]. As such, targeting Sig1R may be a beneficial therapeutic approach to inhibit the progression of BC.

In this context, this study was designed to explore the interaction between Sig1R and β-integrin (adhesion receptors of the ECM), and the process involved in the signaling events associated with the bladder extracellular matrix (BEM). We found that Sig1R was overexpressed in human BC and that this expression was associated with a poor survival rate among BC patients. It was demonstrated that Sig1R interacts with β-integrin, revealing a crosstalk between BC and BEM to promote substance exchange. Our results indicate that Sig1R and β-integrin complexes at least partially participated in BEM-induced proliferation and angiogenesis of bladder cancer cells.

## MATERIALS AND METHODS

### Tissues samples and cell lines

Human BC tissue and adjacent normal tissue specimens were obtained from 40 patients who underwent laparoscopic/open radical cystectomy or transurethral surgical resection at Nanjing First Hospital between September 2015 and September 2020. The study was approved by the Ethics Committee of Nanjing First Hospital and the written informed consent was obtained from all patients. The specimens were collected during surgery and immediately frozen in liquid nitrogen.

Human BC cell lines (T24 and J82) and bladder epithelial cell lines (SV-HUC-1) were obtained from the Chinese Academy of Sciences Cell Bank (Shanghai, China). T24 cells were cultured in the DMEM medium (Gibco, USA), and J82 cells were maintained in the MEM medium (Gibco, USA). SV-HUC-1 cells were maintained in the Ham’s F12K medium (Gibco, USA), and the human umbilical vein endothelial cells (HUVECs) were cultured in the endothelial cell growth medium (Lonza, Switzerland). All media were supplemented with 10% fetal bovine serum (FBS; Gibco, USA), and the cell lines were cultured at 37° C in a humidified atmosphere with 5 % CO2.

### Preparation and characterization of BEM hydrogel

The BEM was prepared from the porcine bladder according to our previously reported protocol [22]. Briefly, a crude sample of the lamina propria was obtained for bladder construction and then placed in 0.25% trypsin/0.038% ethylenediaminetetraacetic acid (EDTA) for 2 h at room temperature. The bladder was then transferred to an ice-cold hypotonic solution consisting of 10 mM Tris-HCl (pH 8.0), 5 mM EDTA, and 10 KIU/mL aprotinin for 24 h. Subsequently, the bladder was transferred to a hypertonic solution containing 50 mM Tris-HCl (pH 8.0), 1.0% Triton X-100 (Sigma-Aldrich, USA), 0.5 M NaCl, 10 mM EDTA, and 10 KIU/mL aprotinin at room temperature for 24 h. The bladder was then transferred to 10 mM Tris buffer (pH 7.6) containing 50 μU/mL DNase I (Sigma-Aldrich, USA) and 1 μU/mL RNase A (Sigma-Aldrich, USA) and was incubated in a shaker for 24 h. BEM was obtained after stirring to remove all cell debris. It was then lyophilized, chopped, and dissolved in 0.1 M HCl with 10% (w/w) pepsin (Sigma-Aldrich, USA), then stirred at room temperature for 48 h to obtain an BEM hydrogel. The BEM hydrogel solution was diluted with 0.2 M AcOH (Sigma-Aldrich, USA) to different concentrations.

### Clone formation assay

T24 and J82 cells in exponential growth phase were inoculated on BEM hydrogel-coated (0 and 20 mg/mL) plates (60mm, Corning, USA) at a final concentration of 1000 cells per dish. Cells were incubated at 37° C and 5% CO2 for 14 days, after which the colonies were fixed with paraformaldehyde (4%) and stained with 1% crystal violet.

### Angiogenesis assay

For the *in vitro* angiogenesis assay, 10 μL of the BEM hydrogel (20 mg/ml) was added to each well of a pre-chilled μ-slide angiogenesis plate (Ibidi, Martinsried, Germany) and then incubated at 37° C for 30 min. When T24 and J82 cell confluence reached 90-100%, the old medium was discarded and replaced with a serum-reduced medium (1% FBS) for 24 h at 37° C. The serum-reduced medium was collected and used as a conditioned medium (CM). HUVECs were collected, resuspended in the CM, and seeded on the immobilized BEM at a density of 10,000 cells/well. The cells were incubated at 37° C for 10 h to allow for tube formation. The length of the tube was analyzed using ImageJ software (Bethesda, MD, USA).

For the *in vivo* angiogenesis experiments, we performed an BEM embolization angiogenesis assay. The CM was mixed with BEM solution (20 mg/ml) in equal volumes and then subcutaneously injected into the two inguinal areas of 4-week-old female nude mice. After 14 days, the stopper was cut off, and the hemoglobin content of the stopper was measured using the Drabkin kit (Sigma-Aldrich, USA). The final hemoglobin concentration was determined using a standard calibration curve after spectrophotometric analysis at 540 nm.

### Construction of stably transfected cells

The Sig1R short hairpin RNA (shRNA) plasmid and Sig1R overexpressing plasmid were purchased from GeneChem (Shanghai, China). A non-targeting shRNA lentiviral vector was used as a negative control. The mature antisense sequence for shRNA used was as follows: 5’-CTGCAGTGGGTGTTCGTGAAT-3’. The cells were seeded onto 12-well plates at a density of 4 × 10^5^ cells/well and cultured overnight at 37° C. The sh-Sig1R plasmid (4 μg) or Sig1R-plasmid (4 μg) was transfected into T24 and J82 cells in the culture medium (2 mL/well) using Lipofectamine 2000 (Invitrogen; Thermo Fisher Scientific, Inc., USA) according to the manufacturer's protocol. To obtain stable cell lines, both Sig1R low-expressing and overexpressing cells were selected with 500 μg/mL neomycin for 14 days. The transfection efficiency was evaluated by western blotting.

### Co-immunoprecipitation (co-IP) and Western blot

Cells were lysed using NP-40 lysis buffer containing protease inhibitors (Roche, USA). Protein A sepharose CL-4B beads (Sigma-Aldrich, USA) were incubated with antibodies against anti-Sig1R, anti-β-integrin, anti-CLIC4, and normal rabbit IgG at 4° C for 4 h (see Supplementary Table 1 for antibody information). Next, the beads were washed three times with NP-40 lysis buffer and then mixed with cell lysates at 4° C overnight. The beads were then washed six times with NP-40 lysis buffer, and the immunoprecipitates were subjected to western blotting.

For western blotting, RIPA lysis buffer (Roche, China) containing a protease inhibitor cocktail was used to extract total protein from the indicated cells. A BCA Protein Assay Kit (Beyotime, China) was then used to determine protein concentration. Equivalent amounts of proteins were subjected to SDS-PAGE, transferred to a PVDF membrane (Millipore, USA), and then blocked with 5% BSA in distilled water at room temperature for 2 h. Subsequently, the membrane was incubated with the diluted primary antibodies at 4° C overnight and then with the HRP-conjugated secondary antibody at room temperature for 2 h. For the primary antibodies (see Supplementary Table 1 for antibody information).

### Proximity ligation assay

To confirm the interaction of proteins *in situ*, a Duolink PLA kit (Sigma-Aldrich, USA) was used according to the manufacturer's instructions. Briefly, the cells were processed, fixed, and incubated with primary antibodies followed by species-specific secondary antibodies conjugated with oligonucleotides (Duolink *In Situ* PLA Probe Anti-Mouse MINUS, DUO92004 and anti-rabbit PLUS, DUO92002, Sigma-Aldrich, USA). The cells were then treated with DNA oligonucleotides and a ligase, and the nuclei were stained with 4’,6-diamidino-2-phenylindole (DAPI). The PLA interaction signal is shown as orange fluorescent dots and analyzed by fluorescence microscopy using a Zeiss Axiovert 200M microscope and Plan Neofluar×40/1.30 Oil (DIC III) objective lens (Carl Zeiss, Jena, Germany). The antibodies used for PLA were anti-Sig1R, anti-CLIC4, anti-β-integrin.

### MTT assay

Cell viability was evaluated using the 3-(4, 5-dimethylthiazol-2-yl)-2,5-diphenyltetrazolium bromide (MTT) assay. Briefly, the cells were seeded on a 96-well plate. At the indicated time points after seeding, cell viability was determined by measuring the absorbance of MTT (Invitrogen, USA) at 490 nm using a microplate reader (MultiscanTMGO, Thermo Fisher, USA).

### Cell cycle

Cell cycle analysis was performed using flow cytometry. BC cells were collected and fixed in 70% ethanol. The cells were then incubated with a propidium iodide staining solution (Beyotime, China) for 30 min in the dark. Samples were analyzed using a FACS Canto II flow cytometer (BD Bioscience, USA).

### Gene set enrichment analysis (GSEA)

In the TCGA dataset, 414 BC samples were divided into two groups based on the expression of Sig1R. To further study the potential function, GSEA software was used to compare the expression profiles of the two groups. The enrichment score and false discovery rate were used to sort the pathways enriched in each phenotype [23].

### Immunohistochemical (IHC) staining and immunofluorescence (IF) analysis

For IHC analysis, the tissues were fixed in 10% neutral buffered formalin, dehydrated, and embedded in paraffin. Sections (5 mm thick) were cut from paraffin embedding blocks. The sections were incubated with primary antibodies at 4° C overnight, followed by incubation with biotinylated secondary antibodies (1:200 dilution, GeneTech, USA) for 1 h at room temperature. The primary antibodies used were as Supplementary Table 1. Subsequently, the tissue sections were incubated with horseradish peroxidase-labeled streptavidin complex for 1 h, stained with diaminobenzidine, and counterstained with hematoxylin.

For the quantitative analysis of IHC, two experienced pathologists examined the stained slides, without prior information about the clinical pathological characteristics of the samples and scored based on the intensity and percentage of positive cells.

For IF, tissues were sliced into 6 μm sections. The cells were cultured on glass slides in a 24-well plate pre-coated with 0.1% gelatin to promote cell adhesion. After fixing in 4% paraformaldehyde, the cells were incubated with primary antibodies at 4° C overnight. Then, the cells were stained with Alexa Fluor® 488, Alexa Fluor® 555, or Alexa Fluor® 647-labeled secondary antibodies (Invitrogen, USA). Subsequently, the cells were stained with DAPI to visualize the nucleus. The primary antibodies used were as Supplementary Table 1.

### Mice studies

Sh-control, Sig1R-KD, or Sig1R-OE T24 cells (approximately 2×10^6^) were resuspended in the BEM (1×10^6^ cells/100 μL) and injected subcutaneously in 4-week-old female nude mice, which were maintained for 5 weeks after the injection. The mice were monitored every 7 days. After 35 days, the animals were ethically euthanized, and the relative data, including weight and the longest and shortest axes of the tumor tissue, were recorded. The caliper was used to calculate the tumor volume every week, and the following formula was used to calculate tumor volume (mm^3^) = (length × width ^2^)/2. All animal procedures were approved by the Institutional Animal Care and Use Committee of the Affiliated Nanjing First Hospital of Nanjing Medical University.

### Statistical analysis

All data were analyzed using SPSS software (version 22.0; SPSS Inc., Chicago, IL, USA) and GraphPad Prism 7 (San Diego, CA, USA). Two-tailed Student’s *t*-tests were conducted to assess the statistical significance between the groups. The survival curves were calculated using the Kaplan-Meier method and analyzed using the log-rank test. Pearson’s correlation was used to estimate the linear relationship between the expression of different genes. All data are shown as the mean ± SD. Statistical significance was set at p < 0.05.

### Data availability

The datasets used and analyzed during the current study are available from the corresponding author on reasonable request.

## RESULTS

### Fabrication of BEM

**Figure 1:**
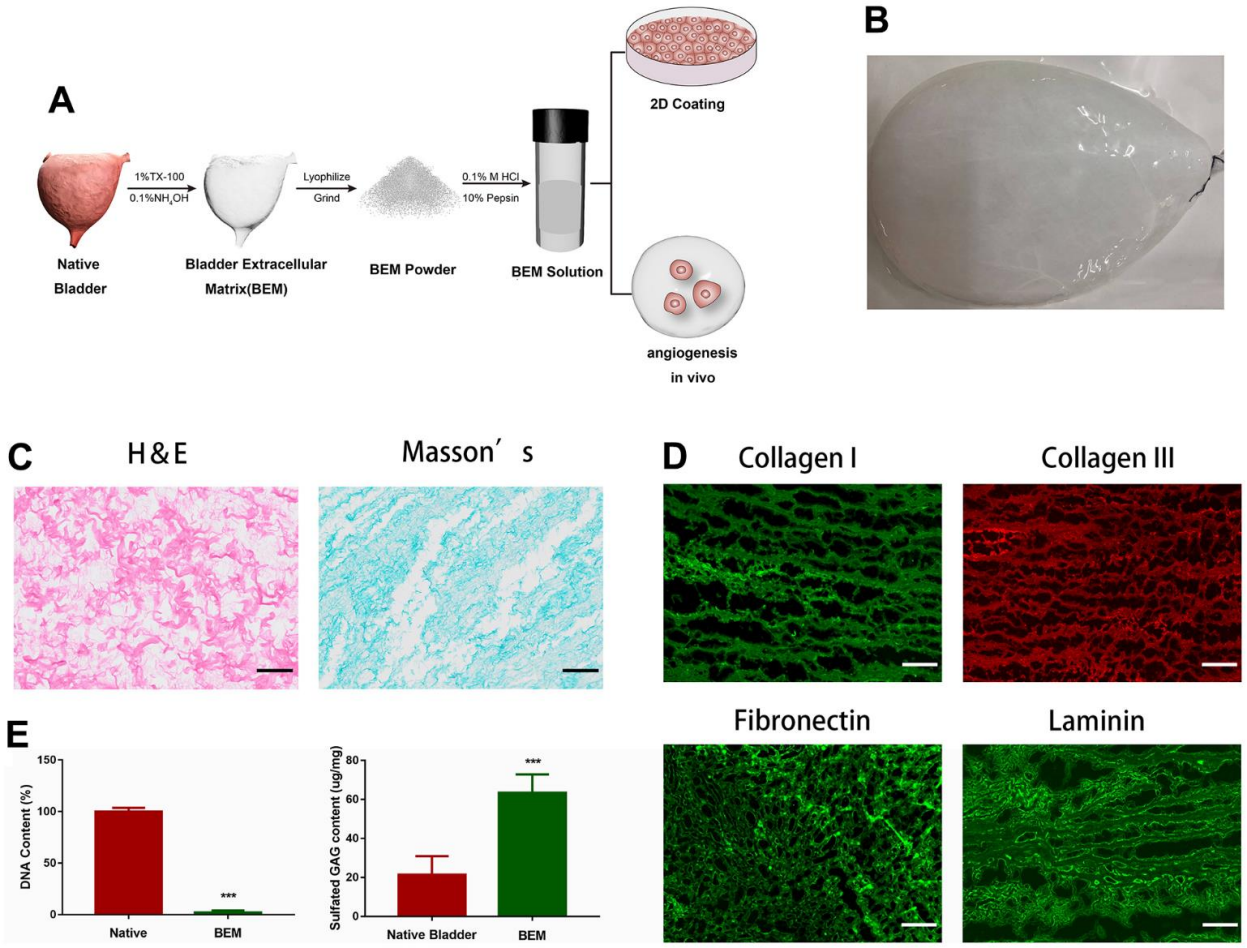
**Preparation and characterization of BEM.** (**A**) Schematic illustration of bladder extracellular matrix (BEM) preparation and application. (**B**) Gross view of a bladder after decellularization. (**C**) H&E and Masson’s staining for BEM, scale bar = 100 μm. (**D**) Immunofluorescence of BEM proteins (collagen I, collagen III, fibronectin, and laminin), scale bar = 100 μm. (**E**) Quantification of DNA and GAG content in native bladder and BEM.

The decellularized bladder, prepared by removing cellular components from the porcine whole bladder and perfusing with a decellularizing solution (1% Triton X-100 and 0.1% NH4OH), is shown in Figure 1A. Decellularization resulted in a transparent bladder tissue (Figure 1B), that was subjected to H&E and Masson’s staining to confirm the removal of cellular components (Figure 1C). Immunofluorescence staining and DAPI nuclear counterstaining of several BEM proteins showed that no DAPI-positive nuclei were detected in the acellular bladder. The main post-BEM components (such as Col I, Col III, fibronectin, and laminin) were decellularized, and they were well preserved in the matrix (Figure 1D). The observed DNA content in the decellularized bladder was the natural bladder tissue (2.2%), which indicated that the most of the cells were successfully removed the bladder tissue. Due to the loss of cell material, glycosaminoglycan (GAG) content increased by 2.96 times (Figure 1E), indicating that the substantial amount of GAG was preserved in BEM after decellularization.

### BEM promotes T24 and J82 cell proliferation and angiogenesis

**Figure 2:**
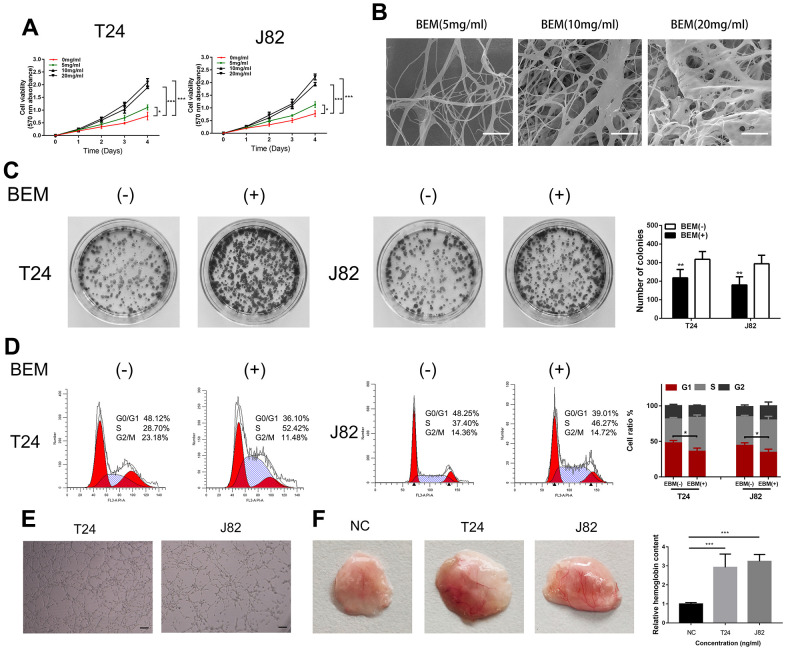
**BEM induces cell proliferation and angiogenesis in bladder cancer cells.** (**A**) Cell viability of T24 and J82 cells incubated with BEM hydrogels (0, 5, 10, and 20 mg/mL) for 4 days was evaluated using the MTT assay. (**B**) SEM images of fibrous collagen structures in BEM hydrogels (5, 10, and 20 mg/mL). Scale bars = 100 nm. (**C**) Cell cycle distribution of bladder cancer cells treated with BEM hydrogels (0 and 20 mg/mL) for 48 h was evaluated by flow cytometry. The percentage of cells in each phase are shown. (**E**, **F**) HUVECs treated with T24 and J82 medium formed capillary-like structures in the, scale bar = 100 μm. *p < 0.05, **p < 0.01, ***p < 0.001.

To study the role of exogenous BEM in the development of bladder cancer and clone formation assays, MTT measurements, flow cytometry, and angiogenesis experiments were performed. The MTT assay showed that BEM enhanced the proliferation ability of both T24 and J82 cells in a concentration-dependent manner (Figure 2A). The internal structure of the BEM hydrogel was analyzed using SEM. Nano-fibrous porous collagen structures were also observed in the BEM hydrogels at concentrations of 5, 10, and 20 mg/ml. We observed that the collagen structure reconstructed in the BEM hydrogel was similar to the structure of natural nanofiber collagen. Considering the results from the MTT assay, 20 mg/ml was selected as the ideal concentration for further experiments (Figure 2B). The clone formation experiments showed that the proliferation of T24 and J82 bladder cancer cells was significantly enhanced after coating with BEM (Figure 2C). Similarly, flow cytometry revealed that after incubation in the BEM, a decrease in the ratio of G0/G1 phase cells was observed in both T24 and J82 cells, whereas the fraction of cells in the S and G2/M phases increased, when compared with that of the cells in the group without coating (Figure 2D). These findings indicate that exogenous BEM promotes the proliferation of bladder cancer. Incubating HUVECs on BEM-coated vascularization slides in the CM resulted in significant angiogenesis (Figure 2E). Similarly, after mixing BEM with the CM and placing it under the skin of nude mice, significant angiogenesis was observed in the stopper after 14 days, suggesting that BEM may promote tumor blood vessel formation (Figure 2F).

### Overexpression of Sig1R correlates with more aggressive clinicopathological features in BC

**Figure 3:**
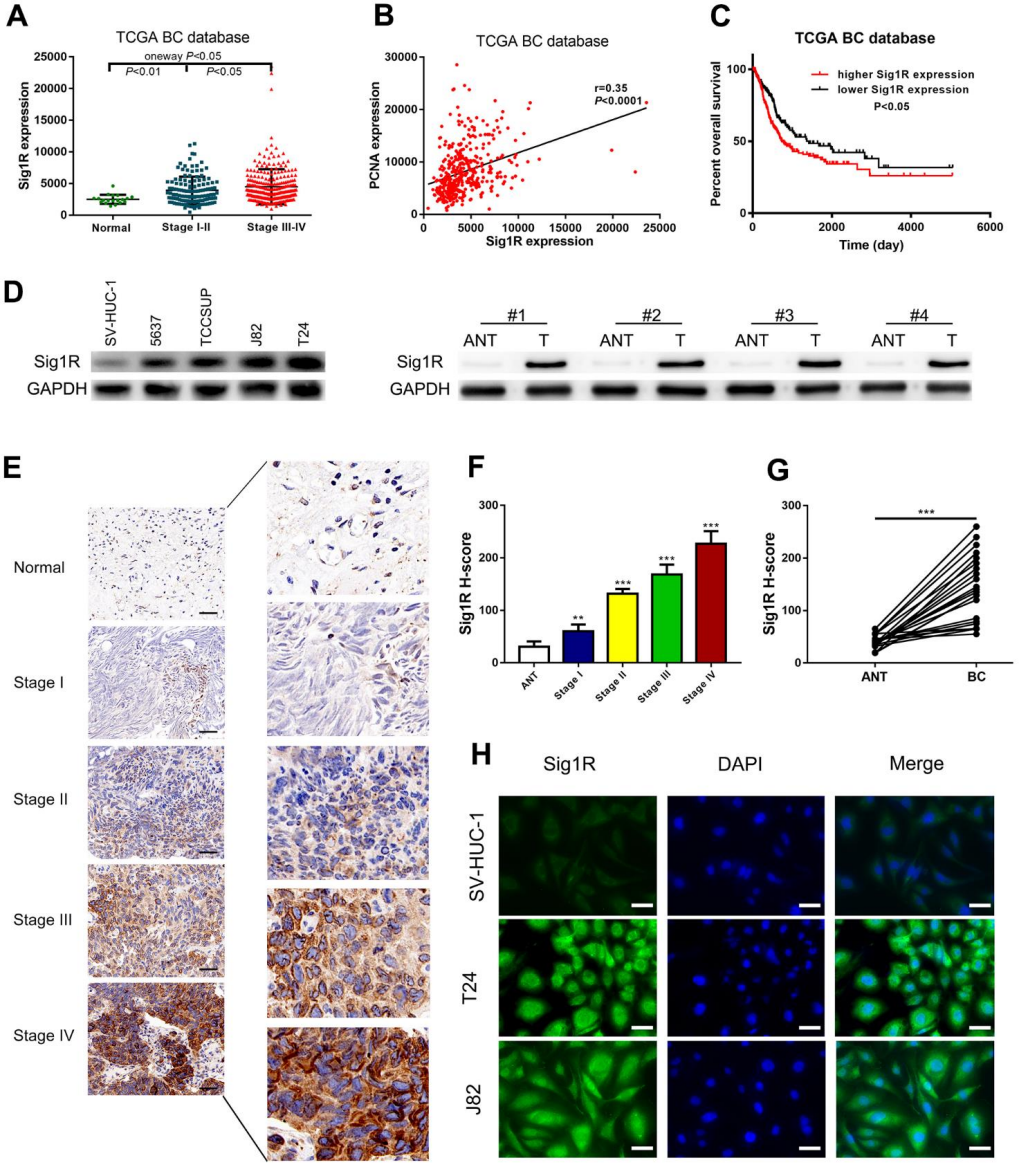
**Up-regulation of Sig1R was closely associated with more aggressive clinicopathological features in bladder.** (**A**) One-way ANOVA analysis showed that Sig1R expression levels significantly correlated with the TNM stage in the TCGA BC RNAseq dataset. (**B**) TCGA BC RNAseq dataset indicated that Sig1R was positively associated with PCNA in bladder cancer tissues. (**C**) The Kaplan-Meier plot of overall survival in the TCGA cohort is shown according to Sig1R expression. (**D**) Human bladder epithelial SV-HUC-1 cell, four bladder cancer cell lines and eight paired bladder cancer tissues, and matched adjacent non-tumor tissues (ANT) were collected to evaluate the Sig1R protein expression by immunoblot. GAPDH was used as a loading control. (**E**, **F**) Examination of Sig1R expression in bladder cancer and normal tissues by IHC using the H-score approach, scale bar = 100 μm. (**G**) Examination of Sig1R expression in 40 paired bladder cancer tissues and ANT by IHC using the H-score approach. (**H**) Sig1R expression and localization in SV-HUC-1 and bladder cancer cell line (T24 and J82) were detected by immunofluorescence, scale bar = 20 μm. **p < 0.01, ***p < 0.001.

We then analyzed the gene expression pattern of Sig1R in the TCGA BC dataset. It was observed that the average expression of Sig1R in BC tissues was higher than that in adjacent normal tissues. Increased Sig1R expression was significantly associated with an increased risk of advanced TNM staging (Figure 3A). In addition, the Pearson correlation test of the TCGA BC dataset showed that Sig1R was positively correlated with PCNA expression, which is a commonly used proliferation marker (Figure 3B). The higher expression of Sig1R was also correlated with significantly lower overall survival (Figure 3C). The analysis of the expression of Sig1R in four BC cell lines, eight pairs of fresh-frozen and 40 pairs of BC tissues, and adjacent normal tissues resulted in similar trends (Figure 3D–3G). IHC was performed using specific anti-Sig1R antibodies to assess Sig1R expression in the BC tissues of 40 patients. Our results indicated that Sig1R is mainly located in the cytoplasm of BC cells. With increasing clinical stages, the expression of Sig1R protein increased significantly (Figure 3E–3G). Additionally, the results showed that the expression level of Sig1R in T24 and J82 BC cell lines was higher than that in normal bladder epithelial cell lines (Figure 3H).

### Sig1R mediates BEM-induced BC cell proliferation and angiogenesis

**Figure 4:**
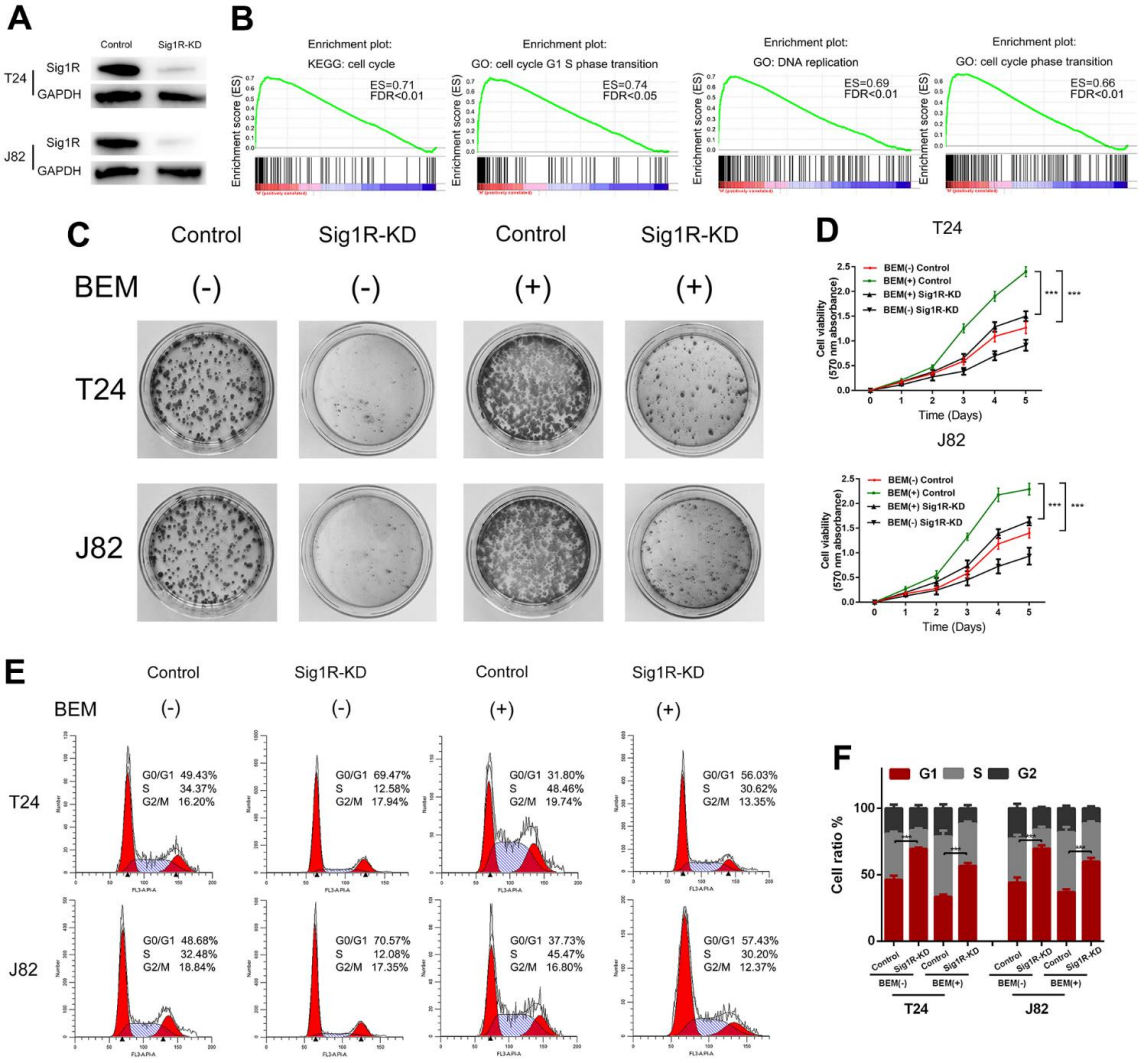
**Sig1R mediates BEM-induced bladder cancer cell proliferation.** (**A**) Sig1R expression levels were examined in Sig1R-KD and control bladder cancer cells (T24 and J82). (**B**) GSEA results showed that Sig1R expression was significantly associated with cell cycle-associated gene signatures, including “cell cycle,” “cell cycle G1–S phase transition,” “DNA replication,” and “cell cycle phase transition.” (**C**) Sig1R-KD and control bladder cancer cells (T24 and J82) were incubated with BEM hydrogel (0 and 20 mg/mL) for 2 weeks and were allowed to colonize. (**D**) The viability of Sig1R-KD and control bladder cancer cells (T24 and J82) incubated with BEM hydrogels (0 and 20 mg/mL) for 4 days was evaluated using the MTT assay. (**E**) the cell cycle distribution of Sig1R-KD and control bladder cancer cells (T24 and J82) treated with BEM hydrogels (0 and 20 mg/mL) for 48 h was evaluated by flow cytometry. (**F**) The percentage of cells in each phase is shown. ***p < 0.001.

We speculated that Sig1R mediates the proliferation and angiogenesis of BC cells induced by the BEM. To define the functional links, lentiviruses were used to knockdown Sig1R expression, followed by western blotting which confirmed the transfection efficiency (Figure 4A). To further explore how Sig1R exerts its carcinogenic effects, GSEA was performed to search gene ontology terms and KEGG pathways enriched in Sig1R high expression samples. Among all the predefined terms, “cell cycle,” “cell cycle G1 S phase transition,” “DNA replication,” and “cell cycle phase transition” were found to be significantly correlated with the expression of Sig1R in the TCGA dataset (Figure 4B). This implicates Sig1R in cell cycle regulation. As expected, Sig1R silencing abolished BEM-dependent cell proliferation (Figure 4C), Similar results were observed in the MTT assay (Figure 4D).

The effects of Sig1R on cell cycle distribution were also demonstrated by flow cytometry. It was found that the fraction of cells in the G0/G1 phase was significantly reduced in the BEM (+) group, whereas the fraction of cells in the S phase and G2/M phase was increased compared with that in the BEM (-) group. Furthermore, compared with that in the BEM (+) control cells, an increase in the fraction of cells in the G0/G1 phase was observed in the BEM (+) Sig1R-KD T24 and J82 cells (Figure 4E, 4F).

**Figure 5:**
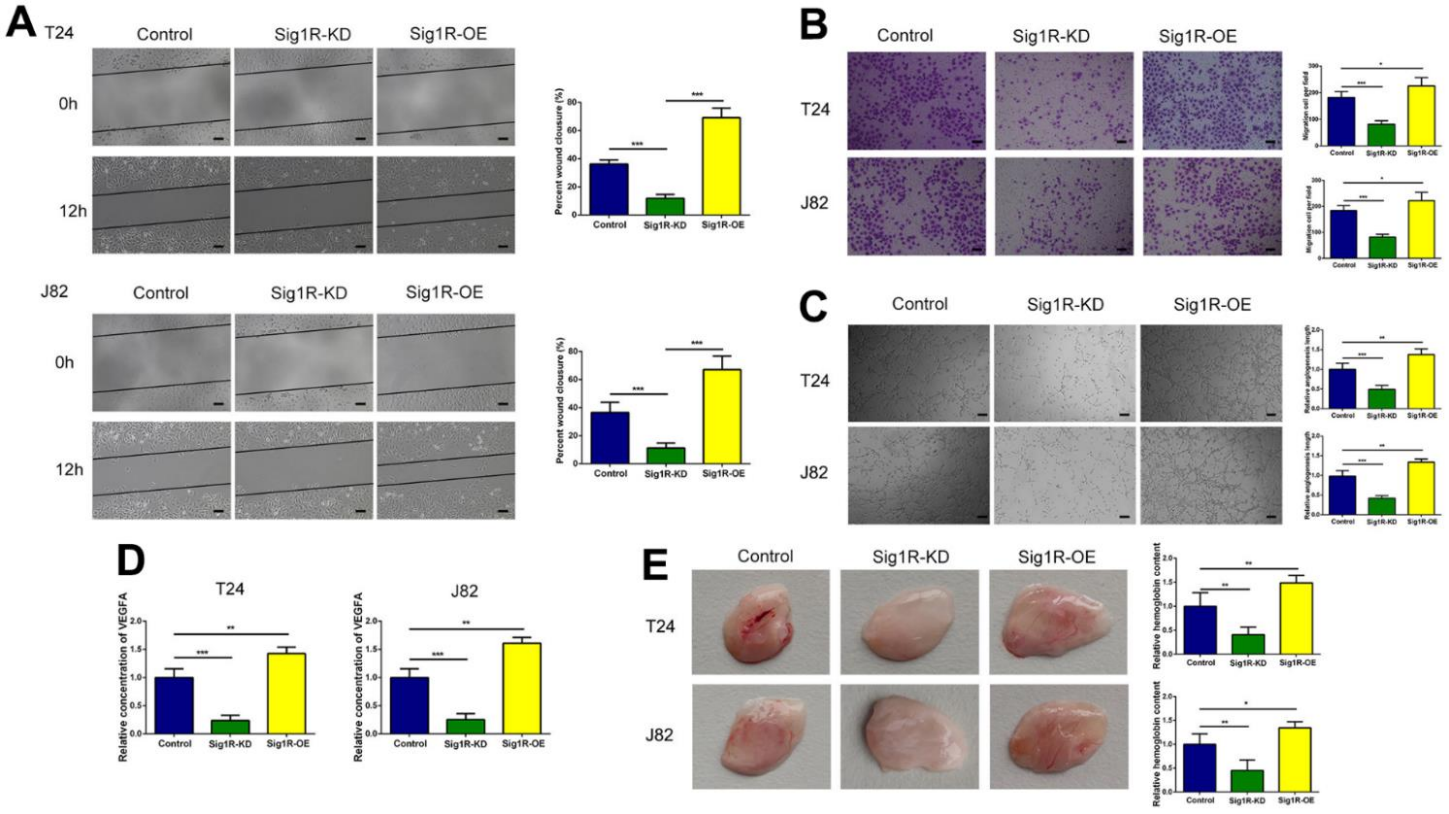
**Sig1R enhanced the crosstalk between bladder cells and HUVECs and promoted migration and tube formation in HUVECs.** (**A**, **B**) Sig1R promoted tumor-induced HUVEC migration according to wound healing and transwell migration assays, scale bar = 100 μm. (**C**) Sig1R promoted tumor-induced HUVEC angiogenesis according to tube formation assays in BEM *in vitro*, scale bar = 100 μm. (**D**) Quantification of VEGFA concentration in CM are shown. (**E**) Sig1R promoted tumor-induced HUVEC angiogenesis according to tube formation assays in BEM *in vivo*. *p < 0.05, **p < 0.01, and ***p < 0.001.

Endothelial cell function is a necessary condition for angiogenesis. Therefore, we analyzed the effects of endothelial HUVEC migration and angiogenesis using an empty vector control, Sig1R knockout, or Sig1R overexpression. We observed that the migration and tube formation potential of HUVECs co-cultured with Sig1R-silenced BC cells was significantly reduced compared with that of the control group cells. Using the same protocol, HUVECs co-cultured with BC cells, showed a stable overexpression of Sig1R, indicating strong migration (Figure 5A, 5B) and tube formation capabilities (Figure 5C). We further determined the dependence of any pro-angiogenesis factors secreted by BC on HUVEC migration and angiogenesis using ELISA. The results demonstrated that compared with that in the normal conditions, HUVECs grown in the Sig1R-KD microenvironment secreted less amount of VEGFA, whereas the VEGFA level in the Sig1R-OE microenvironment was significantly increased (Figure 5D). We then simulated the effect of BC on endothelial cells *in vivo*. Subsequently, we observed significant angiogenesis in the BEM thrombus in the Sig1R-OE group, whereas angiogenesis in the BEM thrombus in the Sig1R-KD group was reduced (Figure 5E). Our results indicate that the overexpression of Sig1R in BC cells may have an indirect effect on the migration and tube formation of endothelial cells.

**Figure 6:**
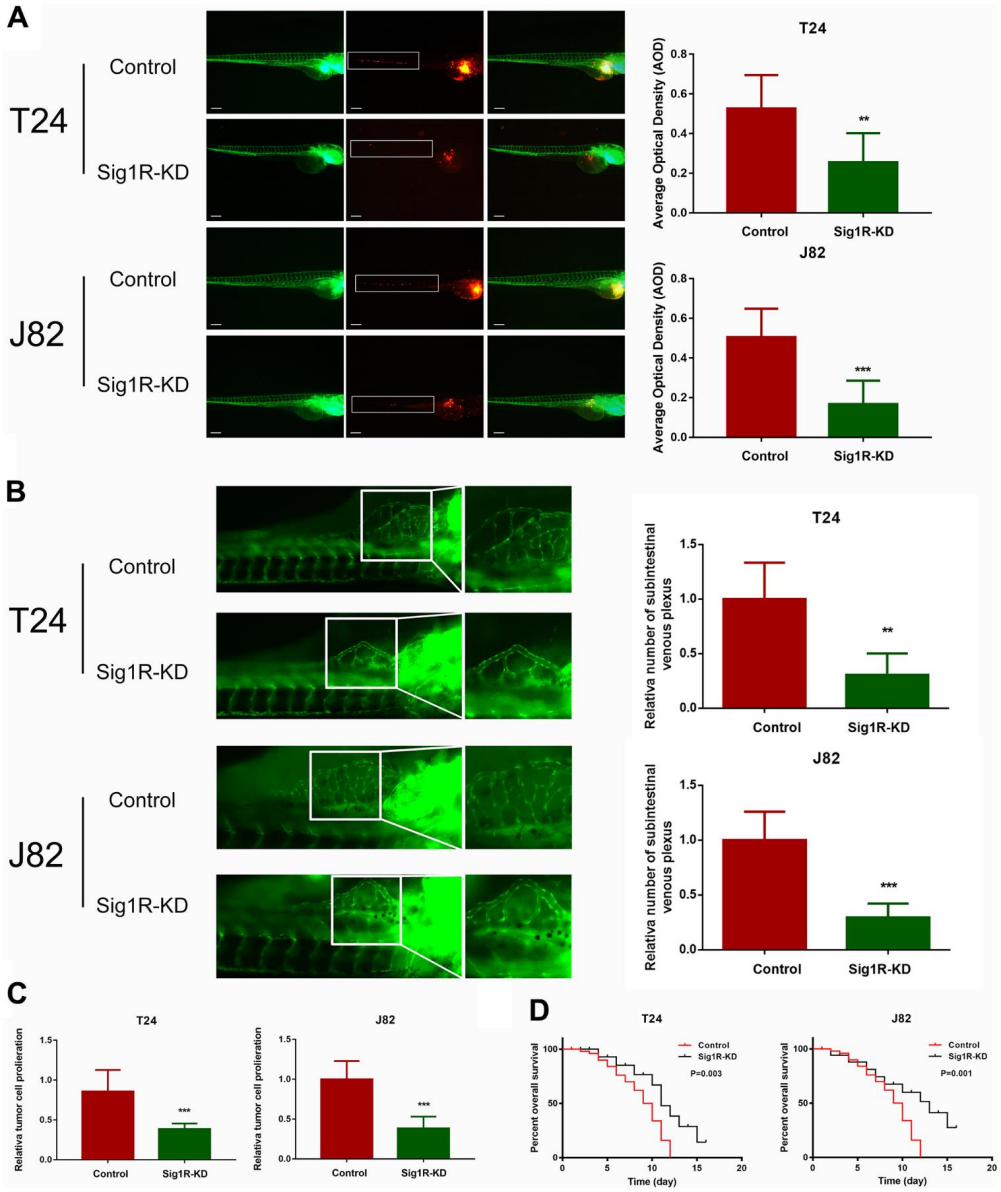
**Sig1R promoted bladder cancer cell invasiveness *in vivo* and reduced overall survival.** (**A**) Dissemination and metastasis of the bladder cancer cells in the zebrafish at 24 h post-injection and quantification of the number of disseminated foci. White boxes indicate disseminated and metastatic tumor lesions, scale bar = 5 μm. (**B**) xenograft in zebrafish for angiogenesis assay. White boxes indicate the regions of pictures shown in the right four pictures; the analysis of the subintestinal venous plexus was conducted. (**C**) The bladder cancer cells (T24 and J82) in the zebrafish at 24 h post-injection. (**D**) Kaplan-Meier analysis of overall survival in the zebrafish post-injection of each group. **p < 0.01, ***p < 0.001.

We further explored whether Sig1R affects BC growth *in vivo*. T24 or J82 cells transfected with Sig1R or an empty vector were transplanted into zebrafish by microinjection. After knocking down Sig1R in T24 and J82 cells, the intensity of red fluorescence in the tail of the zebrafish was significantly reduced compared with that in the control group (Figure 6A). This indicated that tumor invasion and metastasis were effectively blocked. To test the inhibitory effect of Sig1R on angiogenesis and cell proliferation, Sig1R-KD and control shRNA were transplanted into zebrafish embryos. By imaging the development of subintestinal venous plexus, it was found that the silencing of Sig1R reduced the ability of T24 and J82 cells to induce new blood vessel formation (Figure 6B). In addition, we observed that the knockdown of Sig1R reduced tumor proliferation and improved the overall survival rate of the zebrafish (Figure 6C, 6D).

### Sig1R is associated with β-integrin

**Figure 7:**
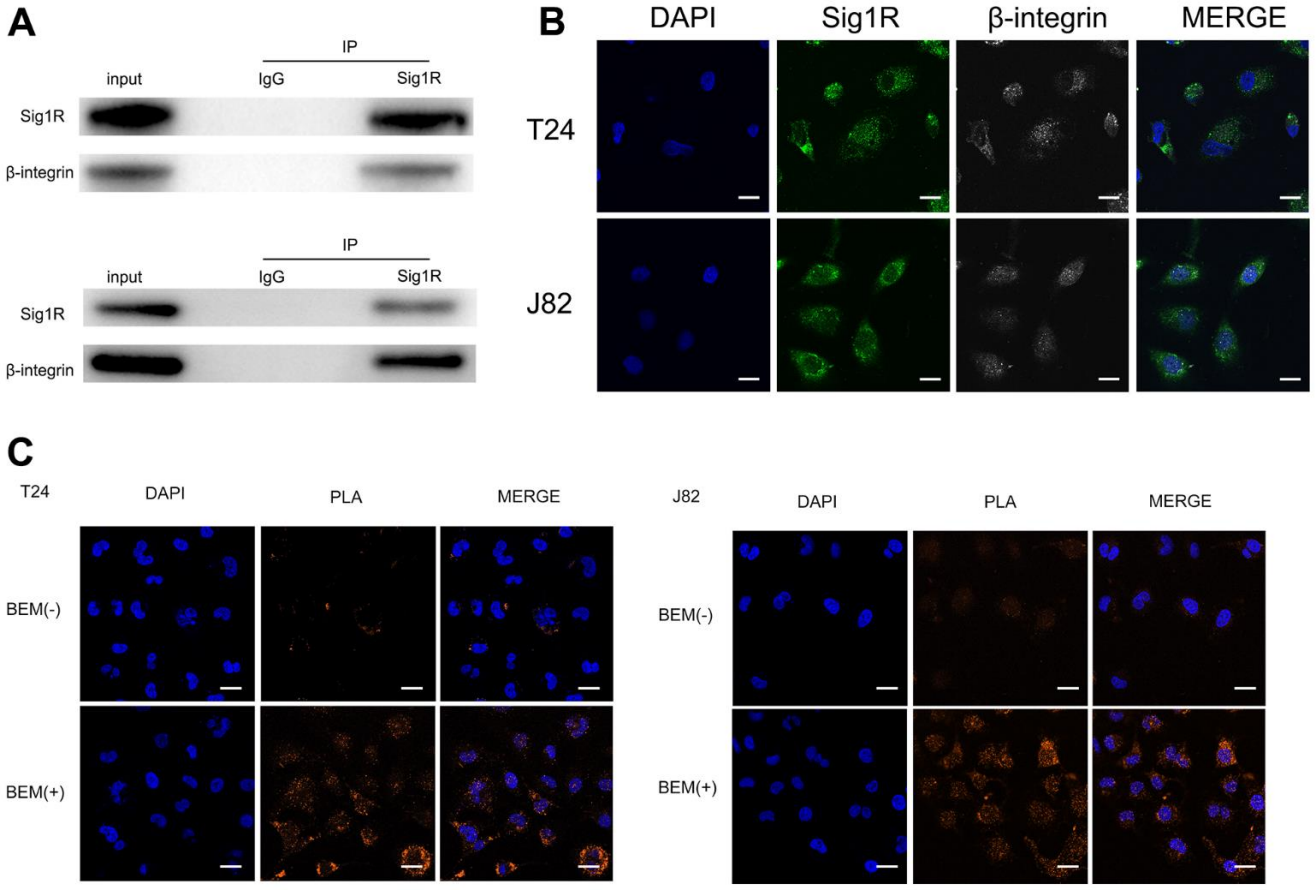
**Sig1R is associated with β-integrin.** (**A**) Co-IP analysis showed the interaction between Sig1R and β-integrin in T24 and J82 cells. Sig1R and β-integrin were immunoprecipitated using antibody against β-integrin. IgG was used as a negative control. (**B**) Immunofluorescence staining analysis showed the colocalization of Sig1R and β-integrin in T24 and J82 cells, scale bar = 20 μm. (**C**) Representative images of Duolink *In Situ* PLA show that there is a direct interaction between Sig1R and β-integrin, and the effect of the two is significantly enhanced after BEM hydrogel coating (orange point), scale bar = 20 μm.

To further explore the mechanism of BEM-mediated bladder cancer invasion and metastasis, we attempted to identify the binding protein of Sig1R. Cell contact with extracellular matrix is mediated by a variety of cell adhesion molecules, β-integrin is the most common adhesion receptor for these cells [24, 25]. Considering that Sig1R and β-integrin may have an important relationship with BC cells in the EBM-mediated process, an immunoprecipitation was performed. Using the Sig1R antibody to detect β-integrin after immunoprecipitation, we confirmed the binding between Sig1R and β-integrin (Figure 7A). In addition, immunofluorescence studies using two protein-specific antibodies showed that Sig1R and β-integrin were co-localized in the cytoplasm of T24 and J82 cells (Figure 7B). The Duolink *In Situ* protein interaction assay was further carried out, where the direct interaction between the two proteins was indicated by the orange signal. We found that in T24 and J82 cells, the interaction signal between Sig1R and the β-integrin complex was mainly located in the cytoplasm and significantly increased in the BEM (+) bladder cancer cells (Figure 7C). These data strongly suggest that Sig1R and β-integrin interact in bladder cancer cells and that BEM can promote the interaction between the two.

Since Sig1R/β-integrin is usually accompanied by the formation of ion-channel protein complexes [18, 21], we screened the ion channel proteins related to Sig1R in the clinical specimens using IHC. The analysis of Sig1R and chloride intracellular channel protein 4 (CLIC4) protein expression in 40 pairs of BC and adjacent normal tissues showed a positive correlation trend (Supplementary Figure 1A, 1B). The correlation analysis of the expression of Sig1R and CLIC4 in the TCGA dataset also produced similar trends (Supplementary Figure 1C) with a high expression of CLIC4 indicating a lower overall survival rate (Supplementary Figure 1D). The above results indicate that Sig1R overexpression is associated with the higher levels of CLIC4 in clinical bladder cancer tissues.

It was confirmed that the action of β-integrin requires agonists to participate in the transport of agonist-regulated β-integrin and CLIC4 [26]. β-integrin and CLIC4 complex are also closely related to the invasion and angiogenesis of cancer cells, and the two are likely to form a complex [24, 26, 27]. Therefore, CLIC4, β-integrin and Sig1R antibodies were used for fluorescence colocalization analysis, and colocalization was detected in the cytoplasm (Supplementary Figure 1E). The interaction signal between CLIC4 and β-integrin was confirmed by the proximity ligation assay (Supplementary Figure 1F). Interestingly, we also detected Sig1R and CLIC4 signals in the cytoplasm (Supplementary Figure 1G). These data indicate that CLIC4 interacts with β-integrin and that Sig1R may also be included in the complex.

### β-Integrin mediates BEM-induced BC cell proliferation, metastatic potential, and angiogenesis *in vivo*

**Figure 8:**
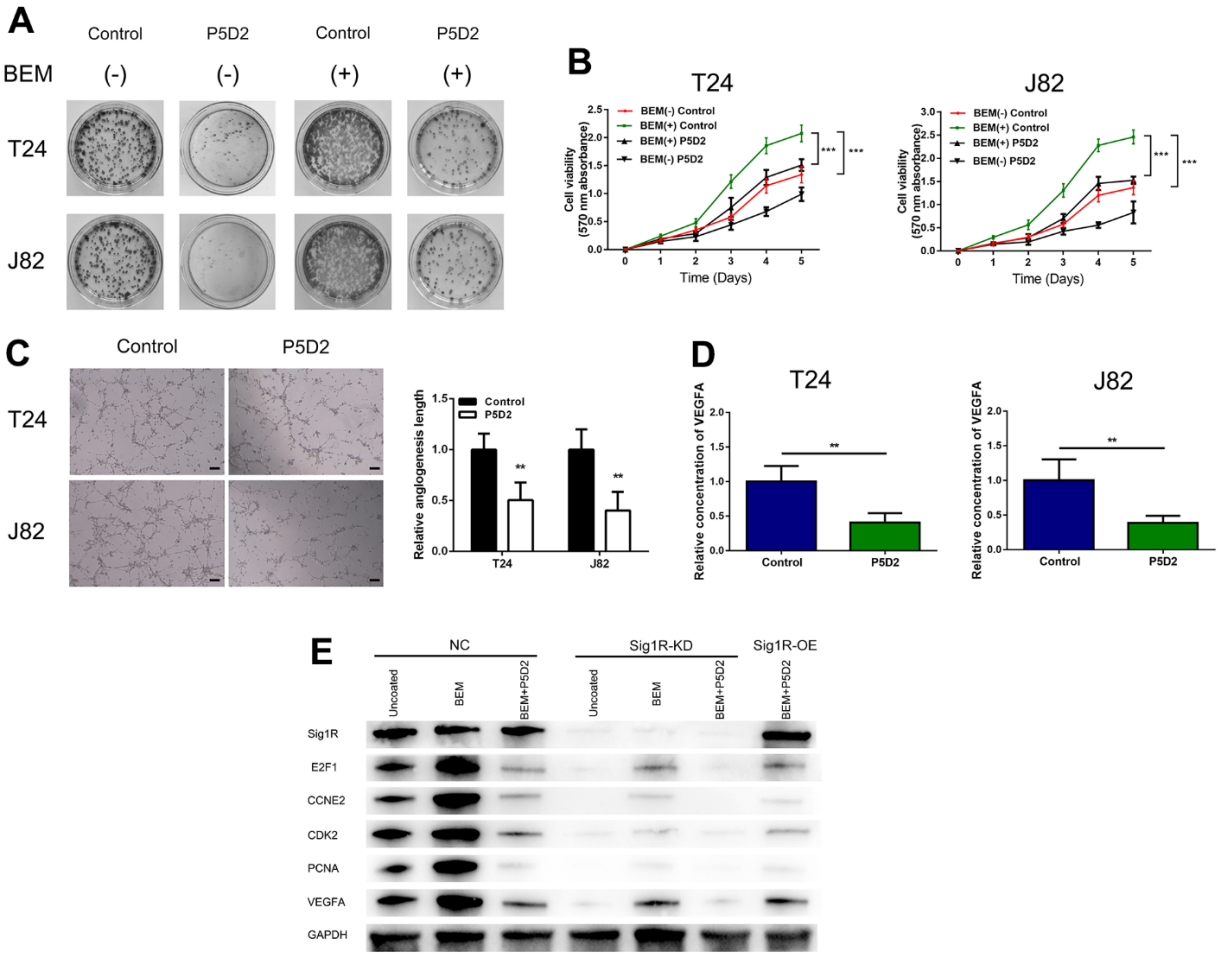
**β-Integrin mediates BEM-induced bladder cancer cell proliferation.** (**A**) P5D2 treatment and control T24 and J82 cells were incubated with BEM hydrogels (0 and 20 mg/mL) for 2 weeks and allowed to form colonies. (**B**) The viability of treated P5D2 and control bladder cancer T24 and J82 cells incubated with BEM hydrogels (0 and 20 mg/mL) for 4 days was evaluated using the MTT assay. (**C**) P5D2 inhibited tumor-induced HUVEC angiogenesis according to tube formation assay in BEM, scale bar = 100 μm. (**D**) Quantification of VEGFA concentration in CM are shown. (**E**) Total protein extracts from NC, Sig1R-KD, or Sig1R-OE K562 cells plated on uncoated or BEM-coated dishes in the presence or absence of P5D2 were separated by SDS-PAGE and immunoblotted with Sig1R, E2F1, CCNE2, CDK2, PCNA, and VEGFA antibodies. **p < 0.01, ***p < 0.001.

Based on above findings, we speculated that β-integrin is associated with the proliferation and angiogenesis of bladder cancer cells induced by the BEM. We treated T24 and J82 cells with the β-integrin inhibitor P5D2 and performed MTT, plate cloning, and *in vitro* angiogenesis experiments. It was observed that the consumption of β-integrin significantly reduced the colony-forming ability and cell viability of the bladder cancer cells induced by the BEM (Figure 8A, 8B). In addition, the angiogenesis assays revealed that knocking out β-integrin reduced the appearance of the tubular structure of HUVECs (Figure 8C). Consistent with these results, a significant reduction in the release of VEGFA was observed (Figure 8D). Overall, we confirmed that β-integrin is involved in the proliferation and angiogenesis of bladder cancer cells induced by the BEM.

We also detected the expression of E2F1, CCNE2, CDK2, and PCNA, which are closely related to the cell cycle. Compared with those in the control group, our results showed that in Sig1R knockout cells, the mRNA expression levels of E2F1, CCNE2, CDK2, and PCNA were significantly reduced. Under BEM conditions, while inhibiting β-integrin and overexpressing Sig1R, no changes were observed in the levels of E2F1, CCNE2, CDK2, and PCNA. This was also observed with the pro-angiogenic factor VEGFA (Figure 8E). These results indicate that the abnormal expression of Sig1R, and its association with β-integrin, affects BEM-mediated BC cell proliferation and angiogenesis by affecting the expression of E2F1, CCNE2, CDK2, PCNA and VEGFA.

**Figure 9:**
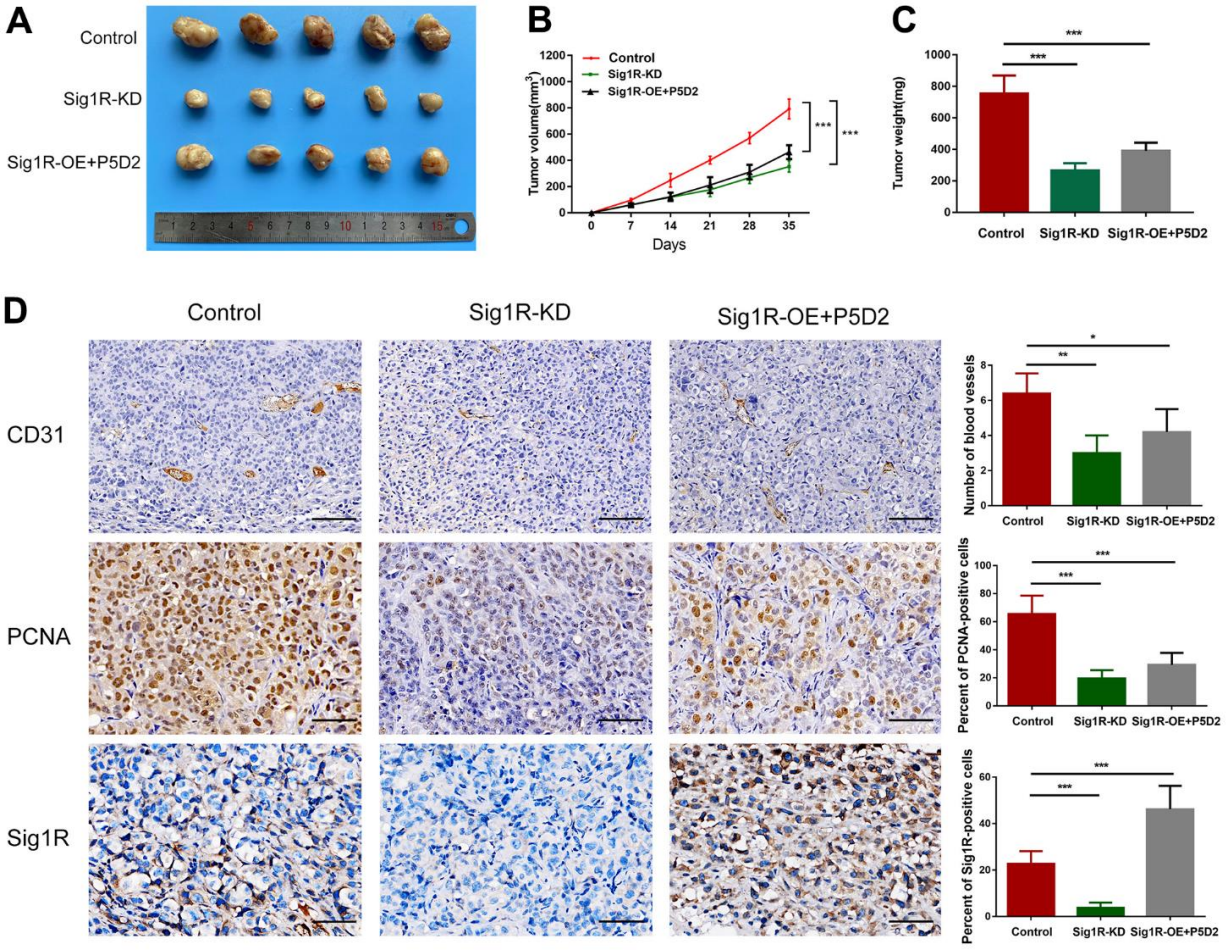
**Sig1R mediates BEM-induced BC cell proliferation *in vivo*.** (**A**) Xenograft model in nude mice. (**B**) Tumor growth curve of Sig1R-KD, Sig1R-KD, or Sig1R-KD T24 cell BC subcutaneous xenograft tumor. (**C**) The weight of dissected xenograft tumors in each group was assayed. (**D**) The IHC staining was performed to detect the expression levels of Sig1R, CD31, and PCNA, in harvested tumor tissues. *p < 0.05, **p < 0.01, and ***p < 0.001, scale bar = 100 μm.

To verify the role of Sig1R/β-integrin in the growth promotion of BC cells *in vivo*, we constructed a xenograft model by subcutaneously implanting T24 cells that knock down or stably express Sig1R into nude mice. All xenograft tumors grew at the injection site, and the mice were euthanized 5 weeks later. As a result, compared with that in the control group, the proliferation of T24 cells in the Sig1R knockdown group was significantly inhibited. The β-integrin inhibitor P5D2 was added to T24 cells overexpressing Sig1R, and no significant increase in the proliferation of the cells was observed (Figure 9A). In addition, the tumor volume and weight of the knockdown and overexpression+P5D2 groups were significantly lower than those of the control group (Figure 9B–9C). IHC analysis showed that compared with that in the control group, the expression of CD31 and PCNA in the Sig1R knockdown group was significantly suppressed. After the application of the β-integrin inhibitor P5D2, even when Sig1R was stably overexpressed, the expression of CD31 and PCNA did not significantly increase (Figure 9D).

## DISCUSSION

Since bladder cancer especially invasive bladder cancer is associated with a poor prognosis and currently has limited treatment options for prolonging survival [28], it is important to analyze the molecular mechanisms that lead to the proliferation and progression of bladder cancer. ECM has unique biochemical and biomechanical properties that promote cancer cell growth, angiogenesis, survival, adhesion, and invasion [25, 29]. Important factors, such as the overexpression of FN in high-grade bladder cancer, have been used as promising biomarkers for the diagnosis and treatment of bladder cancer [30, 31]. In this study, we provided evidence of a link between BEM and bladder cancer cell proliferation and angiogenesis.

Results from this study indicated that there is an interaction between both Sig1R and β-integrin that contributes to the proliferation and angiogenesis of BEM-induced bladder cancer cells. As previously mentioned, it was reported that Sig1R is functionally related to cell cycle control by regulating ion channels and that the regulation of this coupling by sigma ligands can arrest cells at the end of G1 phase [21]. We observed that Sig1R and β-integrin promoted the proliferation of bladder cancer cells by abrogating G0/G1 arrest. Since β-integrins are responsible for regulating cell adhesion [24], it is reasonable that our results demonstrated that it was involved in the progression of BEM-induced bladder cancer.

It is established that angiogenesis is stimulated by growth factors, including VEGFA, which involves endothelial cell proliferation and directional migration into tissues that are nutrient-deficient and then into patent blood vessels [25, 32]. Therefore, we constructed a co-culture system of BC cells and HUVEC to study the indirect effect of Sig1R on HUVEC proliferation, migration, and angiogenesis, and demonstrated that Sig1R increases transendothelial migration and angiogenesis *in vitro*. VEGFA has been shown to be one of the most important growth factors affecting angiogenesis, involving multiple signaling pathways such as ERK [33], tumor necrosis factor-α (TNF-α) [34], phosphatidylinositol-3-kinase (PI3K), and mammalian target of rapamycin (mTOR) [35, 36]. These signaling pathways may cause a subtle cascade of reactions that induce the directional migration of HUVECs [37]. Studies have demonstrated that β1-integrin is involved in VEGFA secretion in a variety of tumors and is associated with promoting invasiveness [38]. We observed that Sig1R and β1-integrin affected VEGFA secretion and promoted the angiogenic capacity of bladder cancer cells. Our study shows that Sig1R/β-integrin in bladder cancer cells are very likely to participate in this process, thereby promoting the progression of BC.

Interestingly, our research also revealed that the signal of Sig1R/β-integrin interacts with CLIC4, which have been detected in high levels in many tumors and regulates the aggressiveness of cancer cells [39–42]. Based on our results, we hypothesize that Sig1R/β-integrin may have formed a complex with the protein channel. This is supported by previous studies in which Sig1R was involved with acid-sensing ion channels [43], voltage-dependent channels [19], and N-methyl-D-aspartic acid receptor [44].

Due to the deep remodeling of the tumor cell microenvironment, the characterization of molecular participants that act between cancer cells and the tumor microenvironment is critical [45]. The ion channels expressed on the surface of tumor cells, like microbial sensors, transmit changes in the structure and function of the microenvironment, allowing the cells to respond accordingly [21, 46], and CLIC4 may be strongly involved in this mechanism and has been characterized as biomarker for many tumors [47, 48]. By building membrane protein platforms with tumor microenvironment receptors, such as β-integrin, CLIC4 was observed to profoundly affect the conduction of tumor metastasis-associated pathways, thereby altering the tumor microenvironment to promote tumor invasion and metastasis [39–42]. Our research strongly suggests that Sig1R, β-integrin, and CLIC4 interact in bladder cancer cells. However, the exact mechanism of tumor progression is still unclear, and further research is needed to determine this mechanism.

In conclusion, our research revealed that the chaperone protein Sig1R interacts with β-integrin, which helps promote cancer cell proliferation and angiogenesis characteristics in response to the BEM microenvironment. Therefore, Sig1R/β-integrin may be a new target for clinical intervention in patients with bladder cancer.

## Supplementary Material

Supplementary Figure 1

Supplementary Table 1
