# Supplementary material for: Sig1R activates extracellular matrix-induced bladder cancer cell proliferation and angiogenesis by combing β-integrin
Source: Aging (Albany NY). 2023 May 16;15(10):4182–201. doi: 10.18632/aging.204721 (PMC10258007; doi:10.18632/aging.204721)
Supplement: Supplementary Figure 1 [file aging-15-204721-s001.pdf]

SUPPLEMENTARY FIGURE

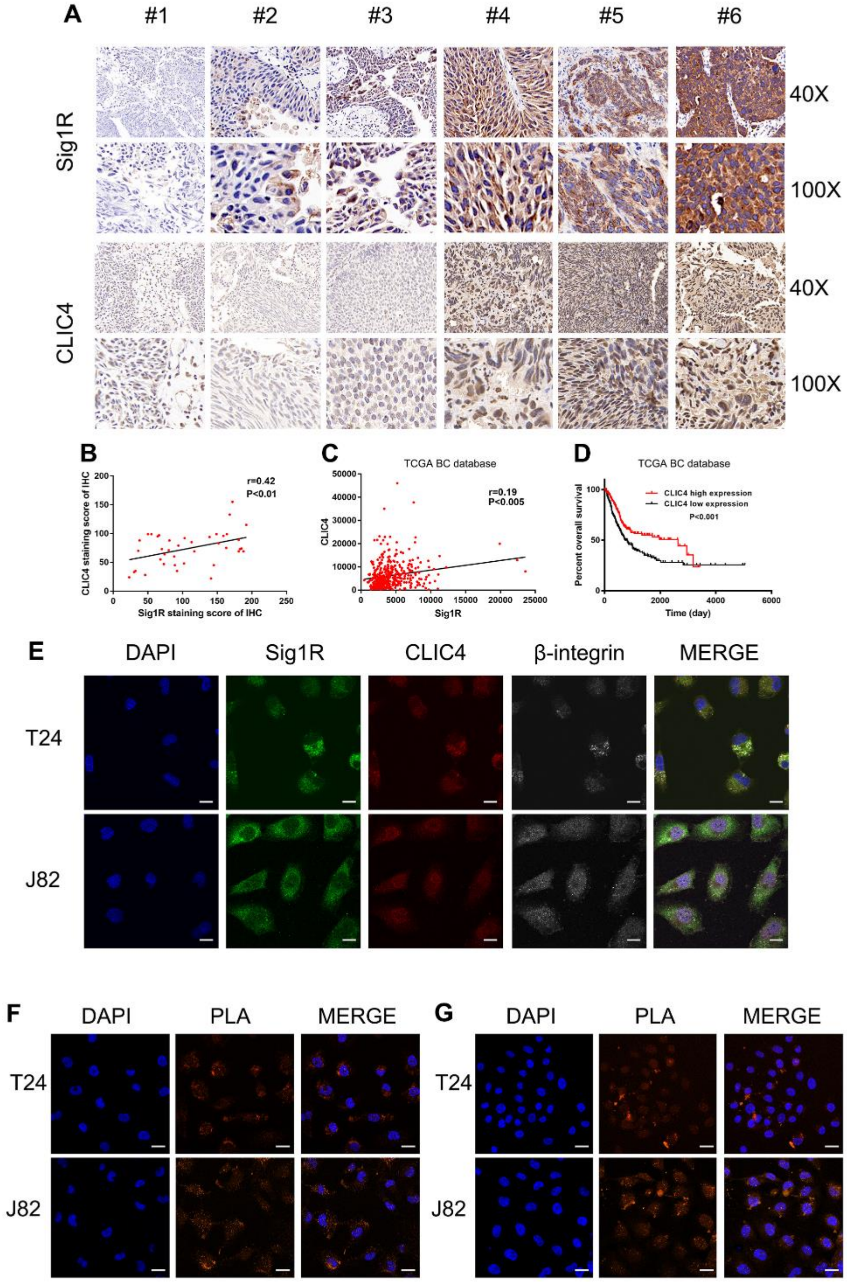

Supplementary Figure 1. The expression of Sig1R and CLIC4 is positively correlated, and they interact with each other. (**A**) Six pairs of representative immunohistochemical pictures are shown. (**B**) Immunohistochemistry was conducted to evaluate the expression of CLIC4 in bladder cancer tissues (n = 40) with low or high Sig1R expression using the H-score semi-quantitative approach. Representative images and corresponding statistical plots (Pearson's correlation coefficients) were presented. (**C**) TCGA bladder cancer RNAseq dataset indicated that Sig1R was positively associated with CLIC4 in bladder cancer tissues. (**D**) The Kaplan-Meier plot of overall survival in the TCGA cohort is shown according to CLIC4 expression. (**E**) Immunofluorescence staining analysis showed the colocalization of CLIC4, β-integrin, and Sig1R in T24 and J82 cells, scale bar = 20 µm. (**F**) Duolink *In Situ* PLA show that there is a direct interaction between CLIC4 and β-integrin (orange point), scale bar = 20 µm. (**G**) Duolink *In Situ* PLA show the direct interaction between Sig1R and CLIC4 (orange point), scale bar = 20 µm.
