# Supplementary material for: Sig1R activates extracellular matrix-induced bladder cancer cell proliferation and angiogenesis by combing β-integrin
Source: Aging (Albany NY). 2023 May 16;15(10):4182–201. doi: 10.18632/aging.204721 (PMC10258007; doi:10.18632/aging.204721)
Supplement: Supplementary Table 1 [file aging-15-204721-s002.pdf]

SUPPLEMENTARY TABLE

Supplementary Table 1. Antibody information.

Name	Application	Manufacturer	Article number	dilution ratio
anti- Sig1R	CO-IP	Santa Cruz	sc-137075	1:50
anti-β-integrin	CO-IP	Abcam	ab24693	1:50
anti- CLIC4	CO-IP	Abcam	ab183043	1:50
anti- normal rabbit IgG	CO-IP	CST	2729	1:50
anti- Sig1R	WB	Santa Cruz	sc-137075	1:500
anti- E2F1	WB	Abcam	ab5391	1:500
anti-CCNE2	WB	Abcam	ab40890	1:1000
anti-CDK2	WB	Abcam	Ab32147	1:1000
anti-PCNA	WB	Abcam	Ab92552	1:500
anti-VEGFA	WB	Abcam	Ab52917	1:1000
anti-GAPDH	WB	Proteintech	60004-1-Ig	1:5000
anti- Sig1R	PLA	Santa Cruz	sc-137075	1:100
anti-CLIC4	PLA	Abcam	Ab183043	1:100
anti-β-integrin	PLA	Abcam	Ab24693	1:100
anti-β-integrin	PLA	CST	34971	1:100
anti-Sig1R	IHC	Santa Cruz	sc-137075	1:100
anti-PCNA	IHC	Abcam	ab92552	1:500
anti-CD31	IHC	Abcam	ab28364	1:50
anti-CLIC4	IHC	Abcam	ab183043	1:100
anti-Col I	IF	Abcam	ab34710	1:100
anti- Col III	IF	Abcam	ab6310	1:100
anti- fibronectin	IF	Abcam	ab24693	1:100
anti- laminin	IF	Abcam	ab44941	1:100
anti-Sig1R	IF	Santa Cruz	sc-137075	1:100
anti- CLIC4	IF	Abcam	ab183043	1:100
anti-β-integrin	IF	BD	553715	1:200
